# Brace treatment for patients with scoliosis: State of the art

**DOI:** 10.4102/sajp.v77i2.1573

**Published:** 2021-10-26

**Authors:** Hans-Rudolf Weiss, Tuğba Kuru Çolak, Manuel Lay, Maksym Borysov

**Affiliations:** 1 Institution of Schroth Best Practice Academy, Neu-Bamberg, Germany; 2 Physiotherapy and Rehabilitation Department, Faculty of Health Sciences, Marmara University, Istanbul, Turkey; 3 Institution of Orthopädie-Technik Lay GmbH, Zell-Barl, Mosel, Germany; 4 Institution of Orttech-plus Rehabilitation Service, Charkiv, Ukraine

**Keywords:** scoliosis, brace application, outcomes, rate of success, cosmesis

## Abstract

**Background:**

Physiotherapy, brace applications or surgery are the treatment options utilised to manage patients with scoliosis. Many different brace applications are used, and the success rates of orthoses vary.

**Objectives:**

Brace applications can have detrimental impacts on the patient leading to physical discomfort, psychological discomfort, and in some instance the use of braces may even be painful. Therefore, future developments in this field should be aimed at improving the success rate and reducing physical distress experienced by the patient while using brace applications. The purpose of this article is to provide recommendations with respect to the most appropriate bracing approach in general.

**Method:**

A narrative review of the scientific literature was carried out to substantiate the statements made in this article.

**Results:**

The most important braces provided for the treatment of patients with scoliosis and the treatment results that can be achieved are presented and discussed, taking into account the most recent systematic reviews. A wide range of success rates have been found for the different brace applications.

**Conclusion:**

Given that brace application may impact the patient leading to physical discomfort and psychological distress, good quality management in brace application for patients with scoliosis is needed to ensure the best possible outcome and the least stressful management.

**Clinical implications:**

Safety in brace application for patients with scoliosis needs improvement. The use of standardised and reliable computer aided design (CAD) libraries and appropriate patient information based on published guidelines is suggested.

## Introduction

Scoliosis is a three-dimensional deformity of the trunk and spine, which in phases of enhanced growth may deteriorate dramatically (Asher & Burton 2006; Goldberg et al. 2002; Kruzel & Moramarco 2020; Landauer, Wimmer & Behensky 2003). There are numerous causes for scoliotic deformities (e.g. congenital scoliosis with malformations of vertebral bodies and/or ribs, neuromuscular scoliosis, scoliosis in mesenchymal disorders, and many other underlying diseases or syndromes) (Chik 2020). About 80% – 90% of all scoliosis cases, however, are of unknown origin and are labelled as idiopathic scoliosis (Asher & Burton 2006).

Adolescent Idiopathic Scoliosis (AIS) or late onset idiopathic scoliosis is the most prevalent type of scoliosis which most commonly presents in adolescent girls (Asher & Burton 2006; Kruzel & Moramarco 2020; Landauer et al. 2003).

Adolescent Idiopathic Scoliosis appears during the pubertal growth spurt and affects girls considerably more (female to male ratio approximately 4:1) than boys. For curves with angles exceeding 40°, the female to male gender ratio is approximately 10:1 (Asher & Burton 2006). Treatment of AIS consists of: (1) physiotherapy, (2) brace application, and (3) spinal surgery (Kruzel & Moramarco 2020).

The first version of the guidelines for conservative scoliosis management were published in 2006 (Weiss et al. 2006) and have been updated recently (Weiss & Turnbull 2020a). During the pubertal growth spurt, the probability of progression can be calculated for each individual case (Lonstein & Carlson 1984). A probability for progression of less than 40% is within the observation range, while the probability for progression of 40% – 60% is an indication for physiotherapy management, and a probability for progression of 60% or more is considered as a bracing application indication (Weiss & Turnbull 2020a).

Brace application in children and adolescents with scoliosis can currently be considered as evidence-based (Weinstein et al. 2013), however, a multitude of different treatment approaches and treatment philosophies exist as outlined below. The most important brace types in use today internationally are the Boston brace and the Chêneau brace. Besides these two main types of braces, night-time braces and soft braces are also available internationally.

### The Boston brace

**FIGURE 1:**
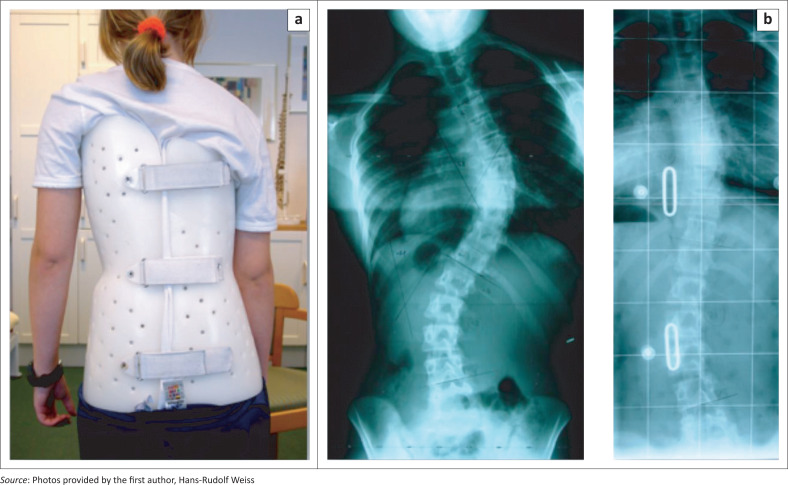
(a) Cast made Boston brace, (b) with a very good in-brace correction in an immature patient.

The Boston brace (Watts, Hall & Stanish 1977) is a thoraco-lumbo-sacral orthosis (TLSO) with dorsal closures usually made of polypropylene (PP) with the inside covered with a polyethylene (PE) foam material for better wearing comfort. The Boston brace may be manufactured individually by use of the plaster cast technique. However, prefabricated Boston brace modules are also available as provided by the Boston Orthotics & Prosthetics located in Avon, MA 02322, USA (Figure 1).

The Boston brace is a symmetric brace usually equipped with pressure pads providing a three-point pressure system aiming to correct the trunk deformity and the spinal curvature. During the pubertal growth spurt, patients are advised to wear the brace for 18–23 hours per day. The flatback deformity usually evident in patients with an idiopathic scoliosis will not be addressed by the application of a typical Boston brace, whilst the lumbar lordosis is usually reduced (Weiss & Turnbull 2020b).

The Boston brace application is supported by a prospective controlled multi-centre study (Nachemson & Peterson 1995), and by a randomised controlled study including an untreated control group (Weinstein et al. 2013). Success rates in both these studies are comparable with 70% and 72% of patients without recorded curve progression (see Table 1).

**TABLE 1:** Rate of success variations as found in literature for brace treatment for patients with idiopathic scoliosis.

Authors	Year	ROS (%)	TIB	BT	Comments
Nachemson and Peterson	1995	70.00	Fulltime	Boston	-
Hanks, Zimmer and Nogi	1988	81.00	Fulltime	Wilmington TLSO	More mature sample
Weinstein et al.	2013	72.00	Fulltime	Boston	Success definition: < 50°
Moreau et al.	2014	67.00	Fulltime	TLSO	Early onset scoliosis
Kuroki et al.	2015	67.70	Fulltime	OMC brace	-
Yamane et al.	2016	23.00 – 58.00	Fulltime	TLSO	No outcome study
Thompson et al.	2017	65.00	Fulltime	TLSO	No outcome study
Xu et al.	2017	75.00	Fulltime	TLSO	-
Minsk et al.	2017	62.00	Fulltime	TLSO	Success definition: < 45°
Harshavardhana and Lonstein	2018	41.00	Fulltime	Boston	Early onset scoliosis
Babaee et al.	2020	64.00	Fulltime	Not specified	Early onset scoliosis
Cheung et al.	2020	60.00	Fulltime	TLSO	-
De Mauroy et al.	2014	95.00	Fulltime	Art brace	-
Bullmann et al.	2004	58.00	Fulltime	Chêneau	-
Weiss and Weiss	2005	80.00	Fulltime	Chêneau	-
Pham et al.	2007	85.70	Fulltime	Chêneau	-
Zaborowska-Sapeta et al.	2011	48.10	Fulltime	Chêneau	-
De Giorgi et al.	2013	100.00	Fulltime	Chêneau	Small curves only
Minsk et al.	2017	85.00	Fulltime	Chêneau	Success definition: < 45°
Weiss et al.	2017	92.00	Fulltime	Chêneau	Curves > 40°
Weiss et al.	2019	92.90	Fulltime	Chêneau	Success definition: < 50°
Weiss et al.	2021	88.00	Fulltime	Chêneau	Curves of 25–40°
Weiss et al.	2021	88.00	Fulltime	Chêneau	Curves of 40° and more
Weiss et al.	2021	96.00	Fulltime	Chêneau	Success definition: < 50°
D’Amato et al.	2001	74.00	Nighttime	Charleston	-
Seifert and Selle	2009	82.20	Nighttime	Chêneau	20–25°
Lee et al.	2012	77.90	Nighttime	Charleston	-
Davis et al.	2019	57.00	Nighttime	Providence	-
Simony et al.	2019	89.00	Nighttime	Providence	Selected cohort

Note: The publications are listed in the order of brace type (Boston or TLSO, Art brace, Chêneau style brace and nightime braces) and by date of publication. Definition of Fulltime may vary (18–23 hours per day); Nighttime is usually defined as 8 hours per night.

ROS, Rate of Sucess; TIB, Time in brace; BT, Brace type.

### The Chêneau brace

The first Chêneau braces were produced by Dr Chêneau in 1976 (Weiss, Rigo & Chêneau 2000), and the first end-results were published by Hopf and Heine (1985). The Chêneau brace is an asymmetric brace addressing different curve patterns individually by inducing a corrective movement.

Originally, the Chêneau brace was made via the plaster cast method. A mould is generated for the patient and filled with plaster in order to develop a plaster model of the uncorrected patient. This plaster model is then modified by cutting off plaster from the prominent regions of the trunk and adding plaster opposite to these regions in order to gain space for the desired corrective movement. The final model is then wrapped by a heated high-density PE sheet which is vacuumed to the model’s surface.

Compression of the patient’s trunk can be avoided when voids opposite to the pressure areas are provided appropriately. During the pubertal growth, spurt a brace wearing time of > 20 hours per day is suggested (Asher & Burton 2006; Kruzel & Moramarco 2020).

**FIGURE 2:**
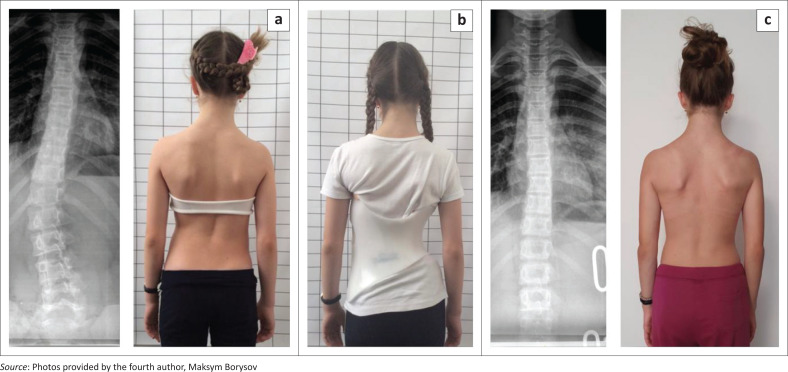
(a) Immature patient with a thoracolumbar curve pattern without the brace, (b) with a good in-brace correction in a Chêneau style brace and (c) a good clinical appearance after 9 months of treatment.

**FIGURE 3:**
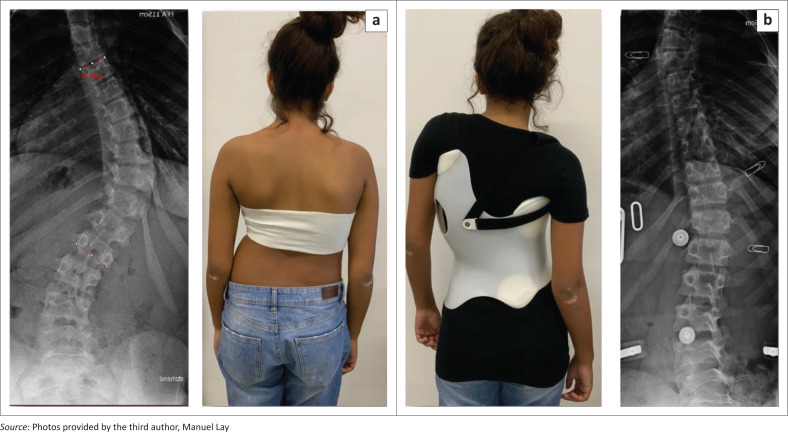
(a) Patient with a severe curve exceeding 60° Cobb angle without the brace and (b) in the special high correction Chêneau style brace (Gensingen brace) for curves exceeding 60°.

Today, Chêneau applications are designed with a computer aided design (CAD (Figures 2 and 3). These braces may be derived from a brace library based on a curvature pattern classification and are virtually adjusted to the patient’s scan on a computer. The file can be used to produce a brace model with a carver, or the brace can be printed (Weiss et al. 2017a).

Chêneau CAD brace series as provided by followers of Dr. Chêneau include the Regnier Chêneau brace (Regnier Orthopaedie GmbH, Achern, Germany), the Rigo System Chêneau or RSC-brace (Ortholutions GmbH & Co. KG, Rosenheim, Germany) and the Gensingen brace (Koob Scolitech GmbH, Neu-Bamberg, Germany, [Weiss 2010]). Other secondary versions exist but may not be standardised. The term ‘Rigo Chêneau’ brace is used by numerous technicians who have followed a course by Dr. Rigo, however these braces in the authors’ experience may vary to a great extent.

### Night-time bracing

The idea that a corrective brace only has to be worn during the night is certainly appealing for those individuals who have to wear a brace. Therefore, an attempt was made to pre-produce corrective braces for night-time use only. Both the Charleston bending brace (Price et al. 1990) as well as the Providence brace (D’Amato, Griggs & McCoy 2001) initially provided promising results specifically for night-time use only. Night-time braces are usually worn for 8 h overnight (Davis et al. 2019; Simony et al. 2019).

**FIGURE 4:**
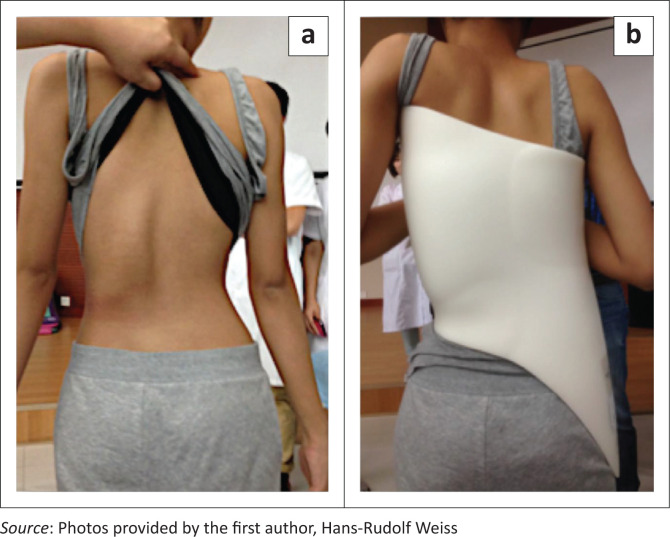
(a) Patient with a thoracolumbar curve, (b) treated with a Providence brace.

Night-time braces are produced by CAD according to hand measurements with the help of measuring tapes and measuring calipers provided by an orthopaedic technician and are sold worldwide (Charleston Brace Company, LLC, Charleston, SC, USA or the Providence Brace by Spinal Technology, Inc., West Yarmouth, MA, USA; Figure 4).

Recent publications have shown contradictory results (Davis et al. 2019; Simony et al. 2019). Therefore, Ruffilli et al. (2021) in their review have stated that a final conclusion about night-time braces cannot be drawn.

### Soft braces

The aim of the application of soft braces has been to increase wearing comfort. There is a lengthy history regarding soft bracing, and the applications as available today may be seen as a revival of soft bracing concepts rather than as recent developments (Weiss 2021).

The SpineCor brace (provided by Spine Corporation Limited, Chesterfield, UK), an application with elastic bands, is widespread and advertises free mobility for the individual in the brace (Coillard, Circo & Rivard 2014). The authors indicated that to initiate and maintain a corrective movement with the SpineCor brace, the brace should be worn full-time during the growing period (Coillard et al. 2014).

**FIGURE 5:**
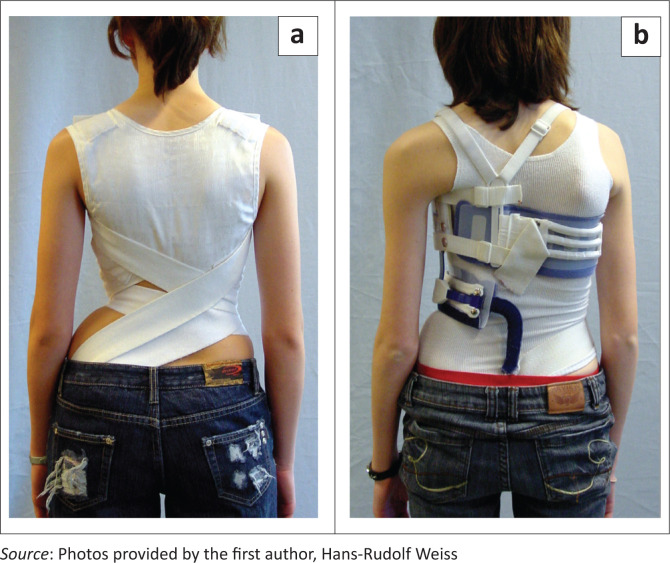
(a) Soft braces as available today. SpineCor for a right thoracolumbar curve and (b) TriaC brace for a right thoracic curve. Clinically no real corrective effect is visible.

The TriaC corset (provided by SPORLASTIC GMBH, Nürtingen, Germany) worn full-time works via a 3-point pressure system (Bulthuis, Veldhuizen & Nijenbanning 2008) and is not suitable for all curvature patterns. While still in distribution, its use is not widespread. Soft braces are naturally not suitable for the treatment of stiff spinal curvatures, as they do not have the restoring force necessary for a sufficient correction (Weiss & Weiss 2005; Figure 5).

**FIGURE 6:**
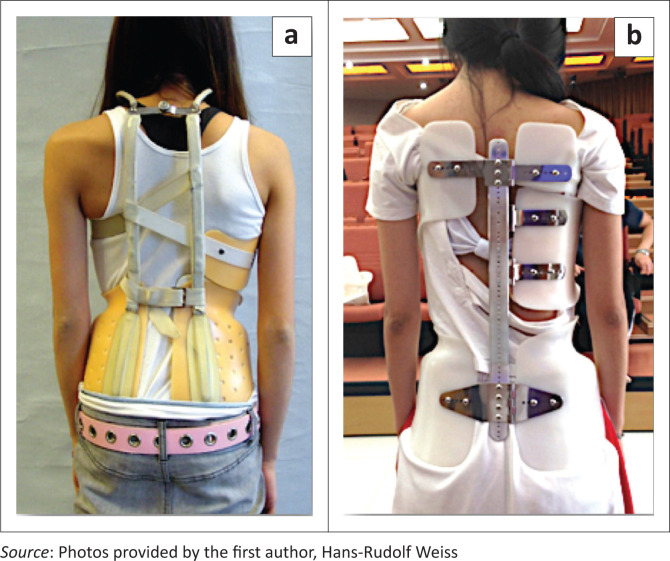
(a) Scoliosis patient in a Milwaukee brace and (b) another in a Lyon brace according to Stagnara. Both braces were made for a right thoracic curve.

There are numerous other brace models, the majority of which are regional variations of existing concepts. From a historical point of view, the formerly widespread Milwaukee brace (Blount 1965) must be highlighted, as well as the Lyon brace (De Mauroy, Lecante & Barral 2011), which still has widespread use in France, but its use is also found occasionally outside of Europe (Figure 6). The further development of the Lyon brace by De Mauroy et al. (2014) has led to a significant improvement in the correction effect and end results. This brace is also currently known as the ART brace (De Mauroy et al. 2014). The Wilmington brace (Hanks, Zimmer & Nogi 1988) also needs to be mentioned. This TLSO is mainly provided in the United States of America.

Regional symmetrical braces, similar to the Boston brace, correct the curve by compressing the trunk and using pressure pads. These types of braces also include the SPORT brace from Italy (Zaina et al. 2011) as well as the Osaka Medical College (OMC) brace (Kuroki et al. 2015). While the SPORT brace, unlike the Boston brace, has front closures, the OMC brace compared to the Boston brace has an axillary support with the aim of improving the corrections of the thoracic curvatures.

This list of different braces for the treatment of scoliosis does not claim to be exhaustive. There are other types of braces available with more regional references, some of which are not listed in the scientific literature or may have a limited range of indications.

In a recent review, it has been shown that braces as applied today have a wide range of results (Weiss & Turnbull 2020b). The success rates ranged from less than 50% to more than 90%. This may be attributed to devices being modelled by plaster cast. Standardisation is not possible with this method of manufacturing. Computer aided design/computer aided manufacturing (CAD/CAM) can be used to standardise brace application. However, the use of CAD/CAM technology alone will not lead to an improvement of the treatment results, but a standardised pattern specific approach addressing the individual curve patterns obviously will (Weiss et al. 2021).

Brace application may adversely impact patients as they may experience physical discomfort, psychological distress, and in some instances the brace may be painful to wear. The aim for future developments should therefore be to improve the success rate and reduce physical discomfort as well as psychological distress in patients who wear braces (Weiss et al. 2007). Besides the influence of a brace on the spinal curve, the possible cosmetic improvements may be important to the patient. The purpose of this article is to provide an overview of the state of the art of scoliosis bracing and to provide recommendations with respect to the most appropriate bracing approach.

## Method

A narrative review of the scientific literature was carried out to substantiate the statements made in this article. The search engines used were: Pub Med, Medline, Embase, Cochrane database, and Google Scholar. Our review analysed outcomes of different brace types used for the management of patients with scoliosis.

The changes in the angle of curvature measured according to Cobb at the end of treatment can be regarded as the most important outcome parameter (Asher & Burton 2006; Kruzel & Moramarco 2020). A variation in the angle of curvature within the range of + or – 5° is generally viewed as unchanged. Changes of 6° and more go beyond the average technical measurement error and are viewed as actual changes. An increase in the Cobb angle is considered progression and a decrease is considered an improvement. Treatment is generally considered successful if the main curvature has not deteriorated by more than 5°.

In addition to the changes in the Cobb angle, it is also important to assess patients with scoliosis as to whether and to what extent changes in the external appearance can be achieved through treatment with a brace. Therefore, studies were also sought in which cosmetic changes were reported.

Search terms used were: (1) scoliosis, brace treatment, rate of success and (2) scoliosis, brace application, cosmetic outcome. Studies published between 1970 and June 2021 are included.

### Ethical considerations

This article followed all ethical standards for research without direct contact with human or animal participants.

## Results

### Results of the Boston brace and other symmetric brace types

The term TLSO describes all underarm braces and the Boston brace is one of these. Usually other TLSO used are more symmetric ‘Boston like’ braces. In many articles the term TLSO is not specified, and the brace used is not documented with a picture. However, usually devices called TLSO use the same principles of correction, namely 3-point pressure application and compression.

As shown in Table 1, in outcome studies the success rates vary between 60% and 81%. However, the study by Hanks et al. (1988) analysed a more mature patient sample than more recent articles (Weinstein et al. 2013; Weiss et al. 2021). Therefore, this article is not comparable to more recent articles and without this article the success rates are between 60% and 75%. As early onset idiopathic scoliosis has a different prognosis than AIS, therefore the articles by Harshavardhana and Lonstein (2018) and Moreau et al. (2014) cannot be considered comparable.

As early as 1995, the prospective controlled multicentre study by Nachemson and Peterson supported the treatment with a Boston brace with high quality evidence. Progression of 6° or more was prevented in 70% of the patients from the treatment group.

The *Bracing for Adolescent Idiopathic Scoliosis Trial* (BRAIST) by Weinstein et al. (2013) utilised a randomised controlled study design and thus provides a high level of evidence. However, the criteria set for success in the study were less strict than in other studies. Treatment is normally viewed as successful if the main curve of scoliosis does not increase by 6° or more. However, the success criterion of the BRAIST study was broader, indicating that if the curvature did not reach or exceed 50°, the treatment was deemed to be successful. Thus, there may be a certain number of patients in this study whose curvature deteriorated by > 5°, but who were still rated as successful as their curvature did not reach the set limit of 50°.

### Results of the Chêneau brace and other asymmetric brace types

In studies in different populations, Chêneau-based applications may lead to better end results when compared to the results of the Boston brace (Minsk et al. 2017; Weiss & Kleban 2015; Weiss et al. 2021). In a pilot study comparing the Rigo-Chêneau brace with the Boston brace in the same population it was found that the Rigo-Chêneau brace with respect to treatment success was superior to the Boston brace (Minsk et al. 2017). Other studies also support the hypothesis that Chêneau style braces may lead to better outcomes than Boston style braces (Weiss & Kleban 2015; Weiss et al. 2021). However the success rates of Chêneau style braces vary to a great extent and may be even worse than the results of the Boston brace or other TLSOs (see Table 1).

The optimal planning and cast modelling of a hand crafted Chêneau brace (Weiss et al. 2000) is a complex procedure to be mastered by the orthopaedic technician. Because of the relatively low prevalence of scoliosis requiring treatment (approx. 0.5%) in the general population (Asher & Burton 2006; Goldberg et al. 2002; Landauer et al. 2003), the opportunity for the orthopaedic technician to gain experience in a short period of time and to constantly improve his or her own skills is limited. This may be one of the reasons why the success rates for asymmetric Chêneau derivates vary to such a great extent.

Another challenge is the varying inclusion criteria. There are prospective cohorts (Weiss & Weiss 2005; Weiss et al. 2021; Zaborowska-Sapeta et al. 2011), retrospective chart reviews (Minsk et al. 2017; Pham et al. 2007), and articles with selective inclusion criteria varying by Cobb angle or maturity. The article by De Giorgi et al. (2013) revealed a success rate of 100%. However, only patients with single curve patterns were included and the average Cobb angle was comparably low (27° at average).

Other articles utilised the Scoliosis Research Society (SRS) inclusion criteria for studies on bracing (Age 10–14 years, Risser 0–2, Cobb angles 25–40°; Richards et al. 2005) and patient cohorts with average Cobb angles between 31° and 33° (Weiss et al. 2019, 2021; Zaborowska-Sapeta et al. 2011). Studies regarding the Chêneau brace outcomes revealed success rates between less than 50% and more than 90% (see Table 1).

While the Lyon brace is a more symmetric TLSO (De Mauroy et al. 2011), the Art brace implements a corrective movement and may be seen to act more like a Chêneau style brace rather than a symmetric TLSO of the Boston style brace (De Mauroy et al. 2014). In their retrospective study, the authors found a success rate of 95%, however the patients treated with the Art brace had correcting casts prior to the application of the Art brace.

### Results of night-time braces

Promising results have been found for the Charleston bending brace as well as for the Providence brace (see Table 1). In a recent article by Simony et al. (2019), a success rate of 89% was reported. The authors reported to have utilised the SRS inclusion criteria for studies on bracing (Richards et al. 2005). In the article, only patients included were with a primarily high in-brace correction (> 60%), while patients with lower in-brace corrections (< 60%) were excluded. Furthermore, in the cohort provided, the Risser stage was not reported, therefore we cannot be sure that all patients have met the SRS inclusion criteria. These challenges were addressed by Potts (2020) in his letter to the editor.

Another non-selective study (Davis et al. 2019) revealed a success rate of 57%. In their retrospective cohort study, Janicki et al. (2007) found a rate of success of the Providence brace treatment of 42%, whilst the success rate of their patients treated with a TLSO was even less.

As early as 1997. Rowe et al. (1997) in their meta-analysis have shown that part-time or night-time bracing is inferior to full-time use. Recent systematic reviews have come to contradictory conclusions. Whilst Ruffilli et al. (2021) in their systematic review were not able to draw a conclusion about the effectiveness of night-time bracing with the Providence brace, Costa et al. (2021) in their systematic review found that in principle there was no difference between part-time and full-time outcomes of brace management. The authors included the study by Simony et al. (2019), although this is a selective analysis of patients with high in-brace corrections. As Simony et al. (2019) did not provide patient data with in-brace corrections of < 60%, their article was not eligible to be included in a systematic review on outcomes of different braces. Therefore, because of the selection bias, the conclusions from the review by Costa et al. (2021) are not justified.

### Results of soft braces

Coillard et al. (2014) in their randomised controlled study found a success rate of 73.1% for their patients treated with the SpineCor in a cohort with curves between 15° and 30° and a Risser stage between 0 and 2. These results were not confirmed in independent high-quality studies (Guo et al. 2014; Weiss & Weiss 2005; Wong et al. 2008) – one study a prospective controlled design (Weiss & Weiss 2005) and two randomised controlled designs (Guo et al. 2014; Wong et al. 2008). In all three articles, SpineCor treatment had a success rate significantly lower than that of rigid orthoses. For other soft braces, according to our review, there are no data available with SRS comparable inclusion criteria. In the study by Bulthuis et al. (2008), only patients with proven flexibility during a bending x-ray were included.

### Cosmetic outcomes of brace treatment

**FIGURE 7:**
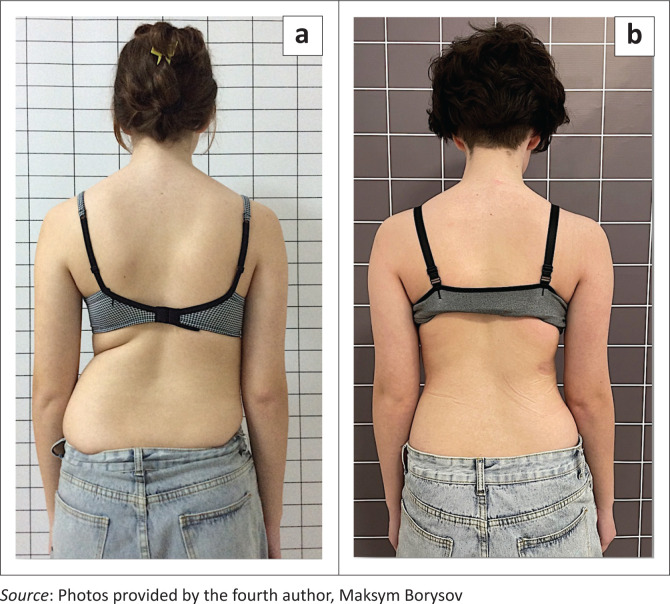
(a) Patient with a significant curve, (b) with a full clinical correction after treatment with a high impact Chêneau-style brace.

**FIGURE 8:**
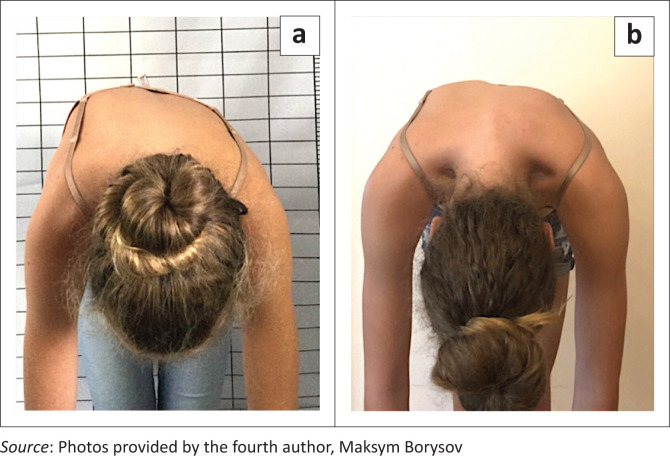
(a) Immature patient with a significant rib hump, (b) with a clear improvement of the rib hump as the intermediate result after 12 months of treatment with a Gensingen brace.

Studies on brace application seem to focus exclusively on the Cobb angle, although this may be of minor importance for patients with AIS. In this largest group of patients with scoliosis, serious health problems are the exception, even in untreated patients (Asher & Burton 2006; Kruzel & Moramarco 2020; Weinstein et al. 2003; Weiss et al. 2016). Therefore, future studies should place an emphasis on which braces are able to positively influence the trunk asymmetry. One parameter for measuring trunk asymmetry is the angle of trunk rotation (ATR) (Bunnell 2005). However, this value only describes the trunk asymmetry when the trunk is bent forward. Even if this value improves only slightly, the improvement in trunk asymmetry in the upright position may be clearly visible (Figures 7 and Figure 8; Weiss et al. 2021). An improvement in trunk asymmetry can be evaluated by using reliable surface topography measurements (Rothstock et al. 2020). Future studies should place more emphasis on cosmetically important clinical parameters especially in patients with AIS because for patients living with idiopathic scoliosis the trunk deformity or their appearance are likely to be more important than the Cobb angle and also because most patients have a rather benign form of scoliosis, which does not lead to serious challenges (Asher & Burton 2006; Kruzel & Moramarco 2020; Weinstein et al. 2003; Weiss et al. 2016).

Obviously, there is a limited number of studies on cosmetic outcomes of brace treatment (Grivas & Vasiliadis 2008; Negrini et al. 2012; Rigo 2003; Weiss et al. 2019; Weiss & Moramarco 2021; Weiss et al. 2021) compared to the numerous studies which use the Cobb angle as an outcome parameter (see Table 1). Grivas and Vasiliadis (2008) in their study found that brace treatment when using a modified Boston brace improved the ATR in lumbar and thoracolumbar curves significantly, while thoracic curves did not improve. In a recent article with a cohort using the Gensingen brace (Weiss et al. 2021), the ATR improved in thoracic as well as in lumbar curves. This indicates that cosmetically important parameters can also significantly improve with high correction braces. For the patient, this may be more important than the changes in the Cobb angle, which in the end did not improve statistically significantly. An aesthetic index was used by Negrini et al. (2012) to demonstrate that brace treatment may improve the trunk deformity in patients with a scoliosis.

## Discussion

Having to wear a brace only overnight or to enjoy full freedom of movement in a corrective device must be perceived by patients with scoliosis as a convincing argument for one or the other brace model. When the attending physician selects a suitable brace, however, the main focus should be on the brace application’s success.

The variability of the results found for all types of braces (Table 1) shows that not one type of brace is fundamentally better than another, after all, the results vary within the individual brace families as well. When considering the best possible care for patients, an averaging of the results, as in the study by Costa et al. (2021), apparently does not support the idea of identifying the best possible treatment approach.

All articles should be read critically by the reader, as even a study utilising the best possible study design could still have shortcomings and limitations. The randomised controlled trial by Weinstein et al. (2013), for example, does not contain any information on the correction effect in the orthosis and the orthosis used is not described in detail, let alone documented in a picture.

The study by Simony et al. (2019) only included patients who achieved a correction effect of 60% and more in the brace. This information was not presented in the abstract and no information was provided in the text regarding the progression or regression of patients with lower correction effects. Nevertheless, the study was found to be eligible to be included in a systematic review with meta-analysis (Costa et al. 2021).

**FIGURE 9:**
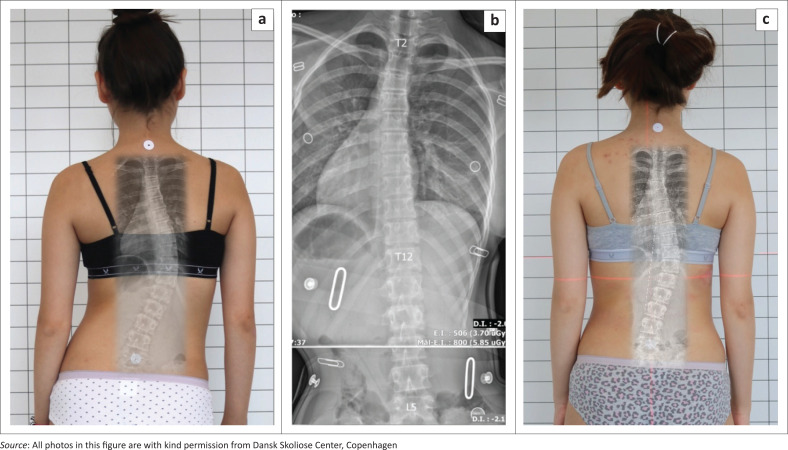
(a) This patient at first was treated with a Providence brace and progressed from 31° to 41° within 6 months before switching to a computer aided design Chêneau brace. (b) In the Chêneau brace, the 41° curve was fully corrected. (c) After 8 months, the compliant patient reduced the curve to 29° and showed improved trunk asymmetry.

As shown in Figure 9, there are patients with their curves at first progressing in a night-time brace and then, after switching to a CAD Chêneau derivate, showing improvement during the management with the new brace. This shows that a progression in a less effective brace is not the end of the road for patients, and will not automatically lead to surgery. These patients, however, certainly would benefit more from brace treatment when the initial treatment was a CAD Chêneau style brace of higher quality.

For those affected, three questions arise: (1) which brace and what wearing time would provide the best chances of success, (2) which brace will have a positive effect on torso deformity, and (3) which brace is the smallest and most comfortable? A brace that allows complete freedom of movement is certainly the most comfortable for patients, but the following question arises: How can a brace achieve the corrective effects necessary in the treatment of scoliosis with complete freedom of movement?

What can be deduced from this narrative review is that the name of the brace alone does not necessarily provide information regarding the chances of success of the treatment. However, Table 1 shows that the best results are achieved from braces that produce a corrective movement. These braces are asymmetrical and there are corresponding free spaces in relation to the pressure zones, which make the corrective movement possible. However, these restorations must correspond to the individual curvature pattern as accurately as possible.

Treating different curvature patterns with different corrective movements in the brace and also in three dimensions is a very complex matter with numerous potential mistakes. Therefore, applying an asymmetrical brace, which provides reliable and good quality results according to the curvature pattern of the patient, using standardised algorithms is recommended. This complex fitting can be best managed, with the assistance of computer technology. Such CAD/CAM brace series have been available for 20 years and are constantly being developed further.

In the case of night-time braces, the correction effects are determined with an X-ray examination in the supine position. Therefore, the percentage correction effects in the brace differ significantly from the correction effects in a full-time brace, which are determined while standing. The correction results while standing are usually lower because gravity loads the curvatures in the upright position and relieves them in the supine position. With full time braces of higher quality, correction effects of approximately 50% can be achieved on average, which according to Landauer et al. (2003) promises a final correction if the patient is compliant.

Previously, it was hypothesised that the initially achieved correction in brace applications of patients with scoliosis would regress within 2 years post-treatment (Landauer et al. 2003). Currently when using high-correcting braces, a significant decrease of initial results in the long-term is no longer expected. Aulisa et al. (2017) observed permanent corrections more than 10 years after weaning the patient from the brace, and the follow-up results did not differ significantly from the results achieved immediately after weaning.

The corrective effect in the brace generally decreases with increasing maturity (Aulisa et al. 2019). Since the results of brace application in patients with scoliosis is clearly dependent on the in-brace correction and the brace wearing time (compliance) (Landauer et al. 2003; Rivett, Stewart & Potterton 2014; Van den Bogaart et al. 2019; Xu et al. 2017), the end results with high-quality braces seem to be the best in immature patients, as their curvatures can be corrected more easily.

Studies with patients having larger angles of curvature (> 40°) show that high success rates can also be achieved with high-quality braces (Aulisa et al. 2019; Weiss et al. 2017b, 2021). These results indicate the need for a standardisation of brace applications for patients with scoliosis to ensure that the impairment in the quality of life of the patient while wearing the brace is a worthwhile endeavour for the patient (Weiss et al. 2007).

To avoid over- or undertreatment (wait and see), it should be mandatory for the informing professional to disclose commonly accepted guidelines where patients can easily find their individual prognosis (Weiss & Turnbull 2020a). This would decrease the uncertainty of patients when receiving contradictory advice and could possibly increase the compliance of patients.

### Limitations of this article

This state-of-the-art article gives an overview of the most important types of braces that are used to treat patients with scoliosis. It does not claim to be complete. It is based on the literature of the common databases without the rigour of a systematic review.

## Conclusion

Taking into account that brace application may impact the patient with possible physical discomfort and psychological distress: good quality management in brace application for patients with scoliosis is needed to ensure the best possible outcome and least stressful treatment. The wide variation of success rates as found in the literature (see Table 1) does not seem acceptable for patients when considering how they sacrifice their time and quality of life to wear the brace, sometimes for years. Curve progression that occurs during the pubertal growth spurt in an ineffective brace cannot easily be reversed at a later stage.
